# Selective determination of 2-aminobenzothiazole in environmental water and organic extracts from fish and dust samples

**DOI:** 10.1007/s00216-023-05035-5

**Published:** 2023-11-10

**Authors:** Alberto Moral, Francesc Borrull, Kenneth G. Furton, Abuzar Kabir, Núria Fontanals, Rosa Maria Marcé

**Affiliations:** 1 Department of Analytical Chemistry and Organic Chemistry, Universitat Rovira i Virgili, Sescelades Campus, Marcel·lí Domingo 1, 43007 Tarragona, Spain; 2 Department of Chemistry and Biochemistry, Florida International University, International Forensic Research Institute, Miami, FL 33199 USA

**Keywords:** Homemade mixed-mode ion-exchange sorbent, Clean-up, Selective solid-phase extraction, Fish, Dust, Complex water samples

## Abstract

In the present study, a homemade mixed-mode ion-exchange sorbent based on silica with embedded graphene microparticles is applied for the selective extraction of 2-aminobenzothiazole (NH_2_BT) followed by determination through liquid chromatography coupled to high-resolution mass spectrometry. The sorbent was evaluated for the solid-phase extraction of NH_2_BT from environmental water samples (river, effluent wastewater, and influent wastewater), and NH_2_BT was strongly retained through the selective cation-exchange interactions. Therefore, the inclusion of a clean-up step of 7 mL of methanol provided good selectivity for the extraction of NH_2_BT. The apparent recoveries obtained for environmental water samples ranged from 62 to 69% and the matrix effect from −1 to −14%. The sorbent was also evaluated in the clean-up step of the organic extract for the extraction of NH_2_BT from organic extracts of indoor dust samples (10 mL of ethyl acetate from pressurized liquid extraction) and fish (10 mL of acetonitrile from QuEChERS extraction). The organic extracts were acidified (adding a 0.1% of formic acid) to promote the cation-exchange interactions between the sorbent and the analyte. The apparent recoveries for fish samples ranged from 22 to 36% depending on the species. In the case of indoor dust samples, the recovery was 41%. It should be highlighted the low matrix effect encountered in such complex samples, with values ranging from −7 to 5% for fish and dust samples. Finally, various samples were analyzed. The concentration in river samples ranged from 31 to 136 ng/L; in effluent wastewater samples, from 55 to 191 ng/L; in influent wastewater samples, from 131 to 549 ng/L; in fish samples, from 14 to 57 ng/g dried weight; and in indoor dust samples, from <MQL to 114 ng/g.

## Introduction

The development of sorptive techniques is one of the leading research areas in the field of sample preparation, and solid-phase extraction (SPE) is one of the most used sorptive techniques because of its reproducibility, low cost, and simplicity [1, 2]. However, one of the main drawbacks of this technique is that most commercial sorbents are unable to selectively extract target analytes [3], so there is increasing interest in obtaining selective materials for extraction. Of these sorbents, particular mention should be made of molecular imprinted polymers (MIPs) [4, 5], tailor-made materials prepared using a template molecule during the polymerization, making the material very selective to the template molecule or similar ones. However, their applications are limited by the template. Another group of selective materials are mixed-mode ion-exchange sorbents, which combine reversed-phase and ionic interactions [6]. The selectivity of mixed-mode ion-exchange sorbents is achieved thanks to a clean-up step that uses an organic solvent (e.g., methanol) to disrupt the reversed-phase interactions. Thus, the compounds retained through ionic interactions can be eluted selectively with an acidic or basic eluent, depending on the nature of the retention.

This selectivity can be exploited to clean up the organic extracts obtained from complex solid matrices. SPE is applied after a previous extraction step such as solid–liquid extraction (SLE) or pressurized liquid extraction (PLE) [7–9] to purify the extract and increase the selectivity of the procedure. In most cases, the analytes are transferred to an organic extract (methanol, hexane, ethyl acetate, etc.) together with various interfering compounds, so a clean-up step is needed. The use of an organic loading solution has its drawbacks, since in SPE, the breakthrough is lower than when aqueous solutions are used, which hampers the retention of the analytes. Therefore, to increase the retention capacity of sorbents, organic extracts are usually diluted in water [8, 10, 11] or partially evaporated to reduce the loading volume [8, 12] or the extract is completely reconstituted in an aqueous solution [8, 13, 14]. Only in a few studies, the organic extract is directly transferred to the clean-up step. For instance, Tsuruoka et al*.* [15] developed a method for the determination of amantadine, rimantadine, and memantine in chicken products where the QuEChERS organic extract (15 mL of acetonitrile with 0.1% of acetic acid) was directly cleaned up with a commercial mixed-mode ion-exchange sorbent (Oasis MCX).

The determination of contaminants in complex samples is a challenge that has yet to be solved. Benzothiazoles are one family of contaminants [16, 17] which occur in samples such as water [18–20], sludge [21], fish [22, 23], dust [24–26], and urine [27], among others. The biological activity of benzothiazoles has been applied to develop a wide range of pharmaceuticals (antimicrobial, analgesic, antitumor…) [16, 28, 29]. Focusing in 2-aminobenzothiazole (NH_2_BT), its derivatives have been synthesized due to their biological activity [30, 31]. However, these compounds with biological activity can cause also negative effects, and some studies have already evaluated their effects of exposure on animals [32–34] and humans [35–37].

In the present study, a mixed-mode ion-exchange silica-based sorbent modified with graphene has been evaluated using SPE for the selective determination of NH_2_BT in complex samples such as aqueous environmental water samples or solid samples such as dust and fish. In the case of solid samples, the sorbent was used to clean up organic extracts from complex matrices like fish or dust samples by introducing a SPE clean-up step after QuEChERS or PLE, respectively. All the SPE extracts were then analyzed by liquid chromatography coupled to high-resolution mass spectrometry (LC-HRMS).

## Materials and methods

### Reagents and standards

Ultrapure water was provided by a water purification system (Millipore, Burlington, MA, USA), and “HPLC grade” methanol (MeOH), ethyl acetate (EtOAc), and acetonitrile (ACN) were acquired from Carlo Erba (Val de Reuil, France). “MS grade” MeOH, ACN, and water were also acquired from Carlo Erba. Hydrochloric acid, formic acid, and ammonium hydroxide were acquired from Sigma-Aldrich (St. Louis, MO, USA).

A solid standard of 2-aminobenzothiazole (NH_2_BT), 97% purity, was acquired from Sigma-Aldrich. A stock solution was prepared in MeOH at a concentration of 1000 mg/L and stored at − 20 °C. Working solutions were prepared weekly in a mixture of ultrapure water and MeOH (85/15, v/v) and stored at 4°C in amber bottles in the dark.

### Structure of the sol–gel mixed-mode sorbent

The sorbent used in the present study was synthesized using a sol–gel approach with tetramethyl orthosilicate, methyl trimethoxysilane, octadecyl trimethoxysilane, N-trimethoxysilyl propyl N,N,N-trimethyl ammonium chloride, and 3-mercaptopropyl trimethoxysilane as building blocks. The conditions of the sol–gel synthesis and its characterization have been described in detail in a previous study [38].

**Fig. 1:**
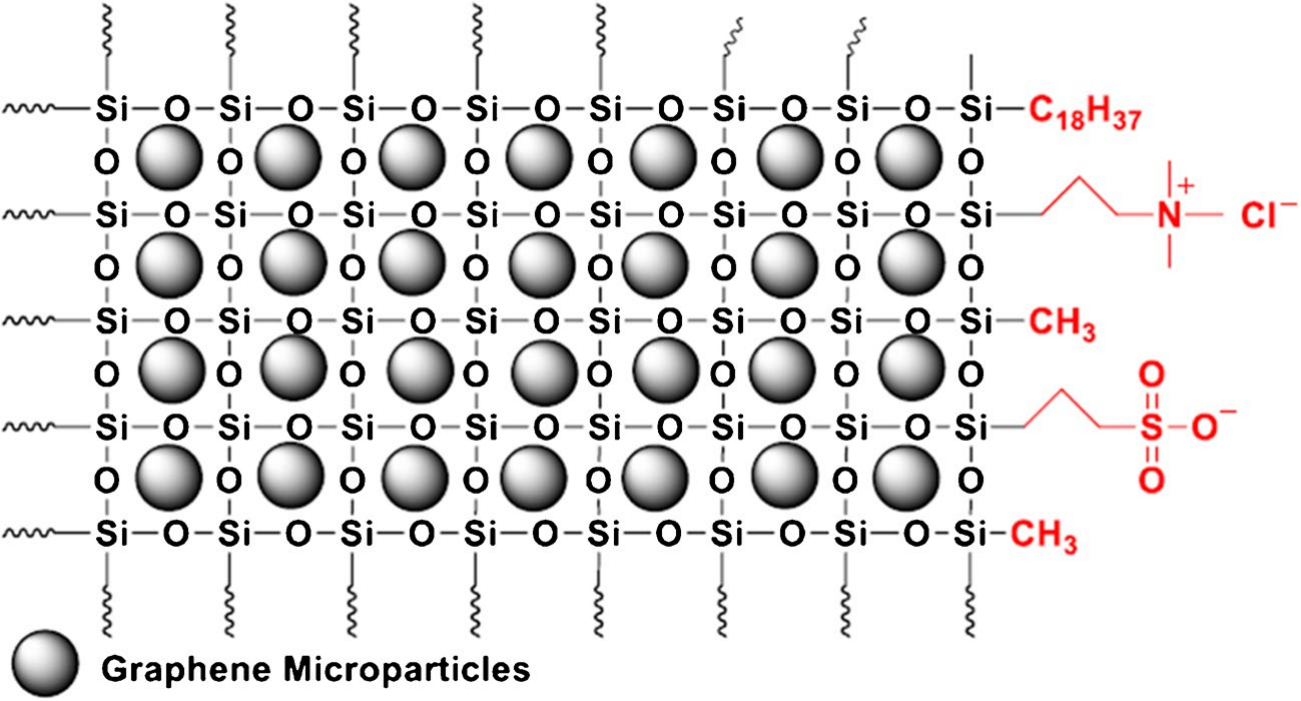
Scheme of the structure of SiO_2_-G-SAX/SCX

The resulting sorbent, SiO_2_-G-SAX/SCX (Fig. 1), is based on a silica modified with graphene microparticles and functionalized with C_18_ chains, quaternary amines, and sulfonic groups, so that it can perform reversed-phase, strong anion- and strong cation-exchange interactions, respectively. Moreover, the addition of graphene microparticles makes π–π interactions possible. The advantage of strong ionic functionalization is that the groups will remain charged at any pH.

### Extraction methods

#### Procedure for environmental water samples

All water samples were collected from the Tarragona region (Spain) and filtered through a 0.45-µm nylon membrane filter (Scharlab, Barcelona, Spain). River samples were collected from the Ebro River. Effluent and influent wastewater treatment plant samples were previously filtered with a 1.2-µm glass fiber membrane filter (Fisherbrand, Loughborough, UK).

A total of 200 mg of the SiO_2_-G-SAX/SCX sorbent was packed in an empty 6-mL SPE cartridge (Symta, Madrid, Spain) between two 10-µm polyethylene frits (Symta). The SPE procedure was performed in an SPE manifold (Teknokroma, Barcelona, Spain) connected to a vacuum pump. With water samples, the first step was to condition the sorbent with 5 mL of MeOH and 5 mL of ultrapure water adjusted to pH 3. Samples of 100 mL of river water or 50 mL of influent and effluent wastewater were adjusted to pH 3 with HCOOH and loaded in the cartridge. The washing step was carried out with 7 mL of MeOH and the elution step with 5 mL of 5% NH_4_OH in MeOH.

The eluted volume was evaporated with a miVac Duo centrifuge evaporator (Genevac, Ipswich, UK) to an approximate volume of 0.1 mL and reconstituted to 1 mL of 0.1% HCOOH in water. The reconstituted extracts were filtered with 0.45-µm PTFE (polytetrafluoroethylene) syringe filters (Scharlab) before analysis.

#### Procedure for indoor dust samples

Dust samples were collected using a vacuum cleaner from houses in the Tarragona region (Spain). The samples were pulverized and sieved (75 µm). Then, they were extracted by pressurized liquid extraction (PLE) with an ASE 350 accelerated solvent extractor system (Dionex, Sunnyvale, USA).

The extraction method was adapted from a previous study [25] in which a 10-mL stainless steel extraction cell was filled with 1 g of diatomaceous earth, 0.10 g, dried weight (d.w.), of sieved dust sample and 1 g of diatomaceous earth. The parameters for the extraction were 1 cycle of EtOAc at 70ºC and 1500 psi for 10 min. Flush volume was 80% of the cell volume, and preheating and purge time were 5 min and 2 min, respectively.

Approximately 20 mL of EtOAc was obtained, which were evaporated to approximately 10 mL with a miVac Duo centrifuge evaporator, and, before the clean-up with SPE, 0.1% of HCOOH was added to the extract.

The SPE cartridge was conditioned with 5 mL of EtOAc and 5 mL of 0.1% HCOOH in EtOAc. The evaporated PLE extract was then loaded in the cartridge and eluted with 5 mL of 5% NH_4_OH in MeOH. The eluted volume was evaporated and reconstituted as in section “Procedure for environmental water samples.”

#### Procedure for fish samples

Fish samples were acquired from markets in Tarragona (Spain), cleaned, frozen, and lyophilized using a miVac Duo sample concentrator with a SpeedTrap freeze-drying system evaporator (Genevac). The extraction method was adapted from a previous study [39]. The samples were ground and sieved (500 µm). A total of 0.10 g of sieved fish sample was placed in a 50-mL centrifuge tube (Serviquimia, Constantí, Spain) with 10 mL of ultrapure water and 10 mL of ACN. The tube was vortexed for 1 min. A QuEChERS extraction salt packet (Standard Method EN15662) (Scharlab) containing 4 g of magnesium sulfate, 1 g of sodium chloride, 0.5 g of sodium hydrogen citrate, and 1 g of sodium citrate was then added to the tube, and it was vortexed for 3 min. The tube was then centrifuged at 4000 rpm for 5 min. The ACN phase was separated, and 0.1% of HCOOH was added before the clean-up with SPE.

The SPE procedure was applied as reported in section “Procedure for indoor dust samples” except that the SPE cartridge was conditioned with 5 mL of the ACN and 5 mL of 0.1% HCOOH in ACN, instead of EtOAc.

### Liquid chromatography–high-resolution mass spectrometry

Chromatographic analysis was performed with an Accela 1250 UHPLC system from Thermo Scientific (Bremen, Germany) with an automatic injector (Accela Autosampler) and a quaternary pump. The chromatographic column used was Acquity UPLC HSS T3 (100 mm × 2.1 mm, 1.8 µm particle size) acquired from Waters (Milford, MA, USA). The mobile phase was a mixture of 0.1% of HCOOH in H_2_O (solvent A) and MeOH (solvent B). The gradient profile started with 0% of B and was increased to 20% within 8 min and then to 100% in 1 min, where it was held for 5 min before returning to the initial conditions in 2 min where it was held for 2 min. The injection volume was 20 µL, the flow rate was 400 µL/min, and the column oven temperature was 45 ºC.

The LC system was coupled to an Exactive Orbitrap mass spectrometer from Thermo Scientific with a heated electrospray ionization (HESI) source and a high-energy collision dissociation (HCD) collision cell for the fragmentation and confirmation of the analytes.

In the HRMS, the signal was acquired in positive polarity. It was optimized in full scan at high resolution (50,000 FWHM) in a mass range of 50–500 m/z. The optimal parameters were a sheath gas flow rate of 40 AU (arbitrary units), an auxiliary gas flow rate of 20 AU, sweep gas 0 AU, spray voltage 4 kV, capillary voltage 37.5 V, tube lens voltage 85 V, skimmer voltage 20 V, capillary temperature 350 ºC, and heater temperature 400 ºC.

Two scan events were used in a range of 50–250 m/z. The first event was a full scan at 50,000 FWHM with 250 ms of injection time. The second was a fragmentation scan at 10,000 FWHM with 50 ms of injection time, applying a collision voltage of 30 eV in the HCD. For quantification, the molecular ion selected was 151.03244 m/z ([M + H]^+^), and it was measured with a mass extraction window of 5 ppm. Two fragments — 109.01145 ([C_6_H_5_S]^+^) and 65.03942 ([C_5_H_5_]^+^) m/z — were used to confirm, as well as the ratio of the molecular ion and its fragments. The confirmation was based on three criteria: the three ions must appear with a signal higher than 10^3^, the ratio between the molecular ion and their fragments must be constant with a variation within ± 30% range, and the retention time must be constant in a ± 0.1 min range.

The instrumental linear range was obtained by analyzing seven solutions, which gave a calibration curve from 0.5 to 500 µg/L (*R*^2^ > 0.995). The instrumental detection limit (IDL) was 0.1 µg/L, which was the concentration that had a signal-to-noise ratio higher than 3 with one of the fragments providing a signal higher than 10^3^. The instrumental quantification limit (IQL) was 0.5 µg/L, the lowest concentration of the calibration curve.

## Results and discussion

### SPE for environmental water samples

NH_2_BT is a benzothiazole, a compound formed by a benzene ring fused with a 1,3-thiazole ring with an amine group, so it can interact with the sulfonic groups in the sorbent through cation-exchange interactions. Moreover, thanks to the electronic delocalization through the fused rings, the compound might interact with the graphene of the sorbent through π–π. As we are interested in exploiting the selectivity of the sorbent, the SPE protocol was studied to exploit the ion-exchange interactions. Based on previous experience [6, 40], the initial protocol consisted of loading the sample at an acidic pH in order to protonate the NH_2_BT, washing with 1 mL of MeOH and elution with 5% of NH_4_OH in MeOH. Nevertheless, this protocol was optimized to further exploit these interactions.

To choose the loading pH, the load of 25 mL of ultrapure water adjusted to pH 3 and pH 6 with a washing step of 1 mL of MeOH was evaluated. Since the %R_SPE_ (SPE recovery, recovery when standard solutions are loaded) at pH 3 (93%) was slightly higher than at pH 6 (85%), pH 3 was selected as the loading pH. Other studies used an acidic pH for the loading solution to determine NH_2_BT in water [18, 19, 40, 41] and urine [42] samples. For instance, Salas et al*.* [40] adjusted the pH of their loading solution to 3 when analyzing environmental water samples with a commercial mixed-mode cationic exchange sorbent (Oasis MCX).

The next parameter to optimize was the washing volume. The volume of MeOH used was increased, evaluating the use of 5 and 7 mL of MeOH. The recoveries of NH_2_BT were not affected by the increasing of MeOH, being 92 and 91% for 5 mL and 7 mL of MeOH, respectively. Since the highest volume of MeOH would provide the highest selectivity possible, 7 mL were selected as the washing volume. The use of ACN as washing solvent was also evaluated, testing 3 and 5 mL. It was discarded since the recoveries of NH_2_BT decreased slightly (83% with 3 mL and 79% with 5 mL).

The use of MeOH was also successfully evaluated for determining benzothiazoles and benzotriazoles with commercial mixed-mode sorbents (Oasis MCX and MAX) [40]. Other studies used water for the washing step with commercial reversed-phase sorbents such as Oasis HLB [18, 42, 43] or Strata-X [19], although water would not have provided the selectivity obtained with MeOH.

The increase in loading volume was also evaluated, increasing from 25 to 100 mL. The recoveries in ultrapure water were similar with values between 87 and 91%. The use of 100 mL of river, effluent wastewater, and influent wastewater was also evaluated. To evaluate the yield of the extraction when working with environmental samples, the apparent recovery (%*R*_app_) was used, which was calculated as the ratio between the experimental concentration of the spiked sample (considering the concentration of the blank sample) and the theoretical concentration. When 100 mL of river sample was percolated, the results were satisfactory; however, with effluent and influent wastewater samples, %*R*_app_ decreased to less than 36%. The loading volume was then reduced to 50 mL, which gave a %*R*_app_ of 69% for effluent wastewater samples and of 62% for influent wastewater samples.

The method for water samples was validated in terms of %*R*_app_ at two levels of concentration, matrix effect, detection and quantification limits, intra-day precision (%RSD, *n* = 4) and inter-day precision (%RSD, *n* = 4), and accuracy, expressed as relative recovery (%*R*_rel_), which was calculated as the ratio between the experimental concentration obtained from the analytical method (considering the concentration of the blank) and the concentration at which the sample was spiked.

Table 1 shows the results of %*R*_app_, spiked at different concentrations depending on the sample volume and complexity; for river samples, it was 0.1 µg/L; for effluent wastewater samples, 0.2 µg/L; and for influent wastewater samples, 0.5 µg/L. As can be seen in Table 1, %*R*_app_ ranged from 63 to 64%, showing good extraction yields. The recoveries were also evaluated at a higher level of concentration, 1 µg/L for river samples, and 2 µg/L for effluent and influent wastewater samples, showing similar results, ranging the recoveries from 62 to 69%.

**Table 1:** Validation parameters for environmental water samples

Sample	%*R*_app_^*^	%ME^*^	Intra-day precision (%RSD, *n* = 4)	Inter-day precision (%RSD, *n* = 4)	%*R*_rel_^*^	MDL (ng/L)	MQL (ng/L)
River	64	+ 1	5	7	105	3	8
Effluent wastewater	63	− 9	3	5	94	9	21
Influent wastewater	64	− 17	9	13	96	14	30

^*^for concentrations in each type of sample, see text

The matrix effect (%ME) was calculated using the formula %ME = (*C*_exp_/*C*_the_ × 100) – 100, where *C*_exp_ is the experimental concentration obtained by spiking a blank sample after SPE (subtracting the signal of the blank) and *C*_the_ is the theoretical concentration. A negative value indicates suppression of the signal, while a positive value indicates enhancement. The extracts were spiked at 10 and 100 µg/L for river and effluent wastewater samples and 25 and 100 µg/L for influent wastewater samples. Table 1 shows the results for the low concentration; in the case of the high concentration, the values were − 1, − 7, and − 14% for river, effluent wastewater, and influent wastewater samples, respectively.

Comparing the results with those of other studies [18, 19, 40, 41] that used polymeric sorbents, it can be observed that the %*R*_app_ are slightly higher than those obtained in the present study. However, the matrix effect was lower with the sorbent applied in the present study because silica sorbents tend to have less capacity than polymeric sorbents. On the other hand, silica sorbents have less non-specific interactions than polymeric sorbents, so the matrix effect is lower. For instance, the recoveries in the present study were lower than the ones reported by Hidalgo et al*.* [41], who obtained a recovery of 83% for NH_2_BT when using a commercial polymeric reversed-phase sorbent (Oasis HLB) to analyze river samples, However, it should be highlighted that the matrix effect was significantly higher (− 25%), compared to the ± 1% in the present study. Salas et al*.* [40] reported similar results to the present study in terms of recovery for influent wastewater samples (%*R*_app_ of 64%); however, the matrix effect was higher (%ME of − 25%) when they used a polymeric mixed-mode ion-exchange sorbent (Oasis MCX). It should be pointed out that in both the studies by Hidalgo and Salas, the amount of sorbent was 500 mg, while in our study, the amount of sorbent was 200 mg. The loading volume should also be considered: it was 1 L in Hidalgo’s study [41] and 100 mL in Salas’ [40]*.*

The method detection limit (MDL) and method quantification limit (MQL) presented in Table 1 were estimated from the instrumental limits (which were based in the signal-to-noise approach) by applying the recoveries obtained for each matrix and the preconcentration factor. MDLs and MQLs were at low ng/L as can be observed in Table 1.

The precision was evaluated in terms of intra-day (%RSD, *n* = 4) and inter-day precision (%RSD, *n* = 4). The intra-day precision was lower than 9%. Meanwhile, the inter-day precision between days was lower than 13% as can be observed in Table 1. Accuracy also was evaluated as %*R*_rel_ at the same levels of concentration used for apparent recoveries; Table 1 shows the results for the low concentration levels, ranging from 94 to 105%.

Several samples were analyzed with the procedures developed. Table 2 shows the concentrations of NH_2_BT obtained. The concentrations for river samples ranged from 31 to 136 ng/L, for effluent wastewater samples from 55 to 191 ng/L, and for influent wastewater samples from 131 to 549 ng/L. Salas et al*.* [40] analyzed similar samples 6 years ago and found lower levels of NH_2_BT in river (31–43 ng/L), effluent wastewater (30–59 ng/L), and influent wastewater (70–160 ng/L) samples. Hidalgo et al*.* [41] also analyzed river samples from the same area but only quantified NH_2_BT in one (31 ng/L). They did not detect the compound in the other three samples (in that study, MDL = 12 ng/L). In samples from other areas, Ao et al*.* [20] found NH_2_BT in a river in Taiwan at significantly higher concentrations (3.9 to 5.1 µg/L). Moreover, Asimakopoulos et al*.* [19] analyzed wastewater samples from Athens but did not detect NH_2_BT (the MDL was at low ng/L).

**Table 2:** Occurrence of NH_2_BT obtained when analyzing different types of complex samples

Sample	NH_2_BT concentration
River water (*n* = 4)	31–136 ng/L
Effluent wastewater (*n* = 4)	55–191 ng/L
Influent wastewater (*n* = 4)	131–549 ng/L
Dust samples (*n* = 4)	< MQL–114 ng/g
Cod (*n* = 3)	14–57 ng/g d.w
Hake (*n* = 3)	21–42 ng/g d.w
Sole (*n* = 3)	24–38 ng/g d.w

### SPE as clean-up for organic extracts from solid samples

**Fig. 2:**
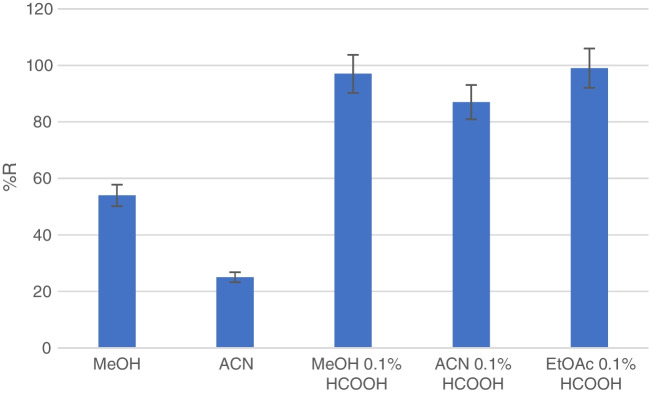
Recoveries obtained when different organic solvents were spiked with NH_2_BT and loaded into the SiO_2_-G-SAX/SCX cartridge

The high retention of NH_2_BT when a clean-up of MeOH or ACN was included made us evaluate the sorbent as a clean-up step for organic extracts. Initially, MeOH and ACN were evaluated as the loading solution. The procedure consisted of a conditioning step with an organic solvent (MeOH or ACN), a loading step with 10 mL of the solvent spiked at 1 µg/L and an elution step with 5 mL of 5% NH_4_OH in MeOH. NH_2_BT was retained, but the recoveries — 54% for MeOH and 25% for ACN — were not very high (Fig. 2). Since the selectivity was achieved thanks to the cationic exchange interactions between the sorbent and NH_2_BT, 0.1% of HCOOH was added to the solvents to protonate the NH_2_BT and enhance these interactions. As can be observed in Fig. 2, the recoveries improved significantly when the loading solvents were acidified and were higher than 87% for all the solvents tested. Moreover, when a mixture water:MeOH (1:1, v/v) was also evaluated, the recovery was 73%. Because the addition of HCOOH is demonstrated to improve recovery, EtOAC with 0.1% of HCOOH was tested, and the recovery was good (99%).

Since the tests with pure solvents provided good results, the sorbent was evaluated as a SPE clean-up for extracts from solid samples like dust or fish.

#### Analysis of indoor dust samples

For dust samples, 0.10 g of pulverized and sieved sample spiked at 1 µg/g was extracted through PLE using the method described in a previous study by Nuñez et al*.* [25]. The approximated 20 mL of EtOAc obtained after PLE was acidified with 0.1% of HCOOH before the SPE clean-up. The %*R*_app_ obtained after the PLE and the SPE clean-up was 30%. To improve the recovery, the PLE extract was evaporated to about 10 mL. Loading 10 mL of 0.1%HCOOH in EtOAc extract increased the %*R*_app_ to 41%, as can be observed in Table 3 together with other validation parameters. So, the evaporation step was included in the procedure. The matrix effect was also evaluated and compared with the %ME obtained without the SPE. When the SPE clean-up was applied, the %ME was + 5%, and when it was not, it was − 62%. Therefore, SPE prevented a significant suppression of the signal. The intra-day and inter-day precision was evaluated as the %RSD (*n* = 3) being lower than 12% in both cases. Accuracy was also evaluated as the %*R*_rel_, being 88%. Dust is a complex matrix, that when analyzed using LC–MS instruments tends to present high matrix effect values. For instance, Du et al*.* [44] determined aryl organophosphate esters in indoor dust and had to apply a clean-up step with Florisil to the extract of ultrasound-assisted extraction. The application of the clean-up made it possible to reduce the matrix effect to levels ranging from − 15 to − 2%.

**Table 3:** Validation parameters for dust and fish samples

Sample	%*R*_app_	%ME	Intra-day precision (%RSD, *n* = 4)	Inter-day precision (%RSD, *n* = 4)	%*R*_rel_	MDL (ng/g)	MQL (ng/g)
Fish	22–37	− 7– + 4	8	15	94–96	5	12
Dust	41	+ 5	6	12	88	6	16

The use of a clean-up step with mixed-mode sorbents for PLE organic extracts has been studied very little and mainly with commercial polymeric sorbents [14, 45, 46]. For instance, Romera-Torres et al*.* [45] used a commercial polymeric sorbent (Strata-X-C) for the clean-up after the PLE of tropane alkaloids from animal feed. They added the acidic moiety directly to the solvent for PLE, and the SPE cartridge was eluted with a solution 3% NH_4_OH in MeOH.

NH_2_BT was determined in indoor dust samples collected from houses in the Tarragona region, the concentration found (Table 2) was between 32 and 114 ng/g in three samples, and in one sample, it was detected below the MQL (16 ng/g). Nuñez et al*.* [25] did not detect NH_2_BT when they used GC–MS to analyze similar samples, probably due to their high MDL (1.5 µg/g). If it is possible to work with a low matrix effect, benzothiazoles should be determined with LC–MS rather than GC–MS due to the higher sensitivity.

When Li et al*.* [26] analyzed indoor dust samples from China, they found NH_2_BT in concentrations ranging from 16.5 to 142 ng/g. Wang et al*.* [24] analyzed indoor dust samples from USA, China, Korea, and Japan. If detected, NH_2_BT was found in concentrations between the MQL (0.5 ng/g) and 31.7 ng/g.

#### Analysis of fish samples

For fish samples, 0.10 g d.w. of sieved fish (cod for the initial trials) was spiked at 1 µg/g, and the QuEChERS extraction described in section “Procedure for indoor dust samples” was applied. Approximately 10 mL of ACN was obtained after QuEChERS. 0.1% of HCOOH was added before the SPE clean-up procedure. The %*R*_app_ obtained after the whole process was 31%, and the matrix effect was only − 1%. This method was compared with another method developed by our research group for determining benzothiazoles in fish using LipiFiltr® for cleaning-up [39]. LipiFiltr® is a cartridge that traps lipids and fats to purify the extract and make it suitable for analysis by GC–MS or LC–MS. The %*R*_app_ for NH_2_BT using the LipiFiltr® clean-up was 51%, and the %ME was + 19%. The %*R*_app_ obtained with the SiO_2_-G-SAX/SCX sorbent was similar to the one obtained with LipiFiltr®, since the use of LipiFiltr® enhanced the signal by 19% while the use of the sorbent SiO_2_-G-SAX/SCX removed practically the matrix effect (− 1%). So, the proposed methodology based on the mixed-mode cation-exchange sorbent provided similar retention and lower %ME than the use of LipiFiltr®. The complexity of fish samples usually provides high matrix effects; for instance, Castro et al*.* [47] reported matrix effects reaching values between − 60 and − 80% when determining organophosphorus flame retardants in mussels when applying a clean-up step with Florisil. Lu et al*.* [48] applied a clean-up with graphene after QuEChERS extraction to reduce the matrix effect to less than 20% when they determined sulfonamides in fish samples.

As can be observed in Table 3, the %*R*_app_ was also evaluated with hake, sole, and other cod samples, which have different fat content, ranging from 22% (sole) to 37% (cod). The matrix effect was also evaluated, ranging from − 7% (hake) to + 4% (cod). The intra-day and inter-day precision were evaluated for all the species, and in both cases, %RSD (*n* = 3) was lower than 15%. In the case of the accuracy, the %*R*_rel_ was higher than 94%.

The use of mixed-mode ion-exchange sorbents for the clean-up of organic extracts from food samples has been studied very little [13, 15, 49]. For instance, one example is the study by Tsuruoka et al*.* [15], who extracted amantadine, rimantadine, and memantine (basic pharmaceuticals) from processed chicken products and performed a clean-up step with a commercial polymeric mixed-mode sorbent (Oasis MCX) after a QuEChERS extraction. They also acidified the organic extract with 0.1% of acetic acid. The elution was also performed with a solution of NH_4_OH in MeOH. The use of a mixed-mode sorbent reduced the matrix effect to remarkably low levels (± 5%).

Three samples of each fish (cod, sole, and hake) from markets in Tarragona (Spain) were analyzed, and the concentration of NH_2_BT was found to be between 14 and 57 ng/g d.w. (Table 2). Trabalón et al. [22] analyzed samples found similar concentration levels, ranging from 11 to 70 ng/g d.w., in similar fish (cod, sole, and hake). Chen et al*.* [23] analyzed marketed fish (bass, billfish, tilapia, and grouper) from Taiwan and did not detect NH_2_BT. Moreover, when Jia et al*.* [50] analyzed mollusks from China, they found concentrations ranging from 53.3 to 93.9 ng/g d.w, slightly higher than the ones in the present study.

## Conclusions

A mixed-mode ion-exchange sorbent based on silica modified with graphene has been successfully evaluated for the selective determination of NH_2_BT. The sorbent was applied for the analysis of environmental water samples (river, effluent, and influent wastewater samples) with an optimized SPE protocol that includes 7 mL of MeOH as a washing step.

When the sorbent was tested as a clean-up of organic extracts from PLE (dust samples) and QuEChERS (fish samples). A total of 10 mL of acetonitrile and ethyl acetate extracts acidified with a 0.1% of formic acid could be loaded through the sorbent without significant losses of NH_2_BT, resulting in a significant decrease of the matrix effect, showing high selectivity.

NH_2_BT was determined in environmental water samples, fish, and dust with low detection and quantification limits. These results are encouraging for the use of this sorbent for the selective extraction of basic compounds from matrices ranging from environmental water samples to complex matrices whose organic extracts need to be cleaned up.
